# Chewing lice (Phthiraptera) species of wild birds in northwestern Turkey with a new host record^☆^

**DOI:** 10.1016/j.ijppaw.2013.07.001

**Published:** 2013-07-30

**Authors:** Ahmet Onur Girisgin, Bilal Dik, Oya Girisgin

**Affiliations:** a Uludag University, Veterinary Faculty, Department of Parasitology, Nilufer, Bursa, Turkey; b Selcuk University, Veterinary Faculty, Department of Parasitology, Selcuklu, Konya, Turkey; c Uludag University, Karacabey Vocational School, Karacabey, Bursa, Turkey

**Keywords:** Chewing lice, Birds, Ectoparasites, Turkey

## Abstract

The goal of this study was to identify the chewing lice species of migratory and non-migratory wild birds in the province of Bursa, which is located in northwestern Turkey, between August 2009 and November 2012. Sixty-eight birds brought to the animal hospital in need of medical intervention, which belonged to 25 species, 20 genera and 15 families in 10 orders, were examined for ectoparasites. To sample for the presence of chewing lice, an insecticide was pulverised on the feathers of each bird over a white piece of paper, and then all of the lice were collected and placed in tubes containing 70% alcohol. The lice specimens were cleared in 10% KOH for 24 h, mounted in Canada balsam and identified using a light microscope. Forty (58.8%) out of 68 birds examined were infested with at least one species of chewing lice, and a total of 29 lice species were found on the birds. This study represents the first documentation in Turkey of 9 of these lice species and also provides the first worldwide record of *Degeeriella nisus* on the Common buzzard (*Buteo buteo*).

## Highlights

• We investigate about chewing lice of migratory and non-migratory wild birds of Turkey.
• Forty (58.8%) out of 68 birds examined were infested with at least one chewing louse species.
• Infestation rate of louse on migratory birds is higher than non-migratory ones, 72.7% and 43.7% respectively.
• Nine lice species are new records for Turkey and one species is a new record of host-parasite association throughout the world.

## Graphical abstract

**Figure:**
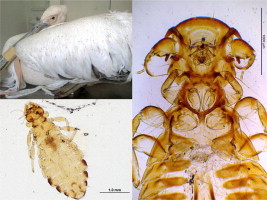

## 1 Introduction

Chewing lice (Ischnocera, Amblycera) are permanent ectoparasites primarily of bird species, and they feed on feathers and skin scales. These lice can be harmful to both domestic and wild hosts, as they deteriorate the quality of the plumage, provoke small holes on feathers (which diminish thermoregulatory capacity), and increase feather breakage (Booth et al., 1993; Kose and Møller, 1999; Vas et al., 2008). To date, over 4000 species of bird lice have been identified worldwide (Price et al., 2003).

Intense lice infestation can potentially produce direct (e.g., hyperkeratosis and feather damage) and indirect (e.g., negative sexual selection) pathological effects for hosts (Lopez et al., 2008; Liebana et al., 2011; Moreno-Rueda and Hoi, 2012). However, infestation was not found to affect nestling growth and survival of broods in American kestrels (Lesko and Smallwood, 2012).

Turkey covers an area of 779,452 km^2^ and has 97 important bird areas (IBAs), which cover a total of 29,978 km^2^ or 4% of the total land area. The country is divided into seven major geographical regions, each with different climates, habitats, flora and fauna. The Bursa province is part of the Marmara Region (40°11′N 29°04′E), which is located along the Marmara Sea, and this province lies along migratory flyways and contains four IBAs (Magnin et al., 2000).

Approximately 110 species of bird lice have been recorded in Turkey, and this number has increased in recent years. Regional studies of multiple wild bird species have been conducted in central and eastern Turkey (Dik, 2010; İnci et al., 2010a; Dik et al., 2011a,b), but few studies have focused on the western region (İnci et al., 2010b). Several studies have also focused on specific hosts, such as Storks (*Ciconia ciconia*) (Dik and Uslu, 2006), Great white pelicans (*Pelecanus onocratulus*) (Dik and Uslu, 2008) and Common blackbirds (*Turdus merula*) (Dik and Dinçer, 2012). Despite these studies, the knowledge of avian louse infestations of wildlife in Turkey remains limited (Inci et al., 2010a), and additional data on the prevalence of chewing lice in wild birds in Turkey is needed (Dik et al., 2011a,b).

Therefore, the objectives of this study were to gather new data regarding the lice species of wild birds in the Marmara Region of northwest Turkey, to increase the knowledge of the geographical distribution of lice found in the study area and to determine the rates of chewing lice infestation in both migratory and non-migratory avian hosts.

## 2 Materials and methods

### 2.1 Study area

This study was conducted at the Animal Hospital of Uludag University in Bursa, Turkey between August 2009 and November 2012. Bursa is a mountainous province with a surface area of 10,891 km^2^ that is covered with natural forest. This region is also generally quite humid (average humidity of 73%) due to the close proximity of the Marmara Sea (Anonymous, 2012).

Due to the unique nature of this animal hospital, members of the public and veterinarians can bring any wild animal in need of medical intervention to the hospital. All of the birds examined in this study were wounded or sick when they reached the hospital. All of the birds studied were kept in separate cages or in limited areas to avoid contamination.

### 2.2 Sampling data

In total, 68 wild birds belonging to 25 species, 20 genera and 15 families in 10 orders were examined for ectoparasites. The identification of birds was conducted according to the guide developed by Heinzel et al. (1995).

Following the identification of the bird species, ectoparasites were collected using a specific product for ectoparasite control that contains a combination of 0.09% tetrametrin and 0.45% piperonyl butoxide. This wide-spectrum insecticide is not harmful to birds when it is pulverised on the feathers over a white piece of paper (Clayton and Drown, 2001). Additionally, the feathers of the wings, the tail and the head/neck region of the birds were separated and inspected (Mey, 2003). All birds were examined immediately following their arrival at the hospital.

The chewing lice collected from the infested birds were transferred to vials containing 70% alcohol and stored in the laboratory until microscopic examination. The protocols for each bird species and the collected lice from all of the infested birds were recorded. At the laboratory, the lice were clarified in 10% KOH for 24 h, mounted on permanent slides with Canada balsam and identified using a light microscope in accordance with the keys developed by Clay (1940, 1958, 1966, 1977), Carriker (1947), Clay and Hopkins (1954), Tandan (1958, 1964), Price and Beer (1963), Tendeiro (1973, 1974), Pilgrim (1976), Clayton (1990), Martín-Mateo (1994), Mey (1998) and Adams et al. (2005). All mounted specimens were stored at the parasitology department laboratories of the Veterinary Faculties of Uludag (Bursa) and Selcuk (Konya) Universities.

### 2.3 Parasitism rate analysis

The infestation prevalence of chewing lice was evaluated for bird families and bird species with a minimum of a single collected individual. The abundance mean and intensity mean level of each species of chewing lice on the avian hosts were determined.

## 3 Results and discussion

Chewing lice were found on 40 (58.8%) of the 68 species of wild birds examined. Fifteen birds (37.5%) were infested with at least two species of lice. Thirty-six species of migratory birds were examined, with 26 (72.2%) presenting infestation. Of the non-migratory birds, 32 species were examined, with 14 (43.7%) presenting infestation.

In addition, a new host-parasite association was found, and 9 species of chewing lice were identified for the first time in Turkey, thereby increasing the geographic distribution of these species (Table 1).

A total of 1,278 lice specimens were collected, representing 523 males, 573 females and 182 nymphs. Some birds had only one or a few lice (e.g., *Passer domesticus*, *Larus cachinnans*), while others presented numerous lice species (e.g., *Pelecanus onocrotalus*, *Platalea leucorodia*). The lice were distributed across two suborders and three families, including suborder Amblycera with families Laemobothriidae and Menoponidae and suborder Ischnocera with family Philopteridae. Identification to the species level was achieved for 30 taxa distributed across 20 genera.

For the family Laemobothriidae, a single species was identified: *Laemobothrion maximum* (Scopoli, 1763).

For the family Menoponidae, five species were identified, including *Ciconiphilus quadripustulatus* (Burmeister, 1838), *Colpocephalum eucarenum* (Burmeister, 1838), *Colpocephalum zebra* (Burmeister, 1838), *Colpocephalum nanum* (Piaget, 1890) and *Piagetiella titan* (Piaget, 1880).

For the family Philopteridae, the following 23 species were identified: *Ardeicola ciconiae* (Linnaeus, 1758), *Ardeicola plataleae* (Linnaeus, 1758), *Coloceras chinense* (Kellogg and Chapman, 1902), *Coloceras hilli* (Bedford, 1920), *Coloceras piageti* (Johnston and Harrison, 1912), *Columbicola bacillus* (Giebel, 1866), *Craspedorrhynchus platystomus* (Burmeister, 1838), *Cuclotogaster heterographus* (Nitzsch [in Giebel], 1866), *Degeeriella fulva* (Giebel, 1874), *Degeeriella nisus* (Giebel, 1866), *Degeeriella rufa* (Burmeister, 1838), *Degeeriella leucopleura* (Nitzsch), *Struthiolipeurus struthionis* (Gervais, 1844), *Falcolipeurus suturalis* (Rudow, 1869), *Philopterus fringillae* (Scopoli, 1772), *Saemundssonia lari* (Fabricius, 1780), *Strigiphilus cursitans* (Nitzsch [in Giebel], 1861), *Goniodes pavonis* (Linnaeus, 1758), *Goniodes dispar* (Burmeister, 1838) and *Ibidoecus plataleae* (Denny, 1842). Some individuals of the genera *Pectinopygus* and *Degeeriella* were also collected but could not be identified due to unsuccessful preparation.

**Fig. 1:**
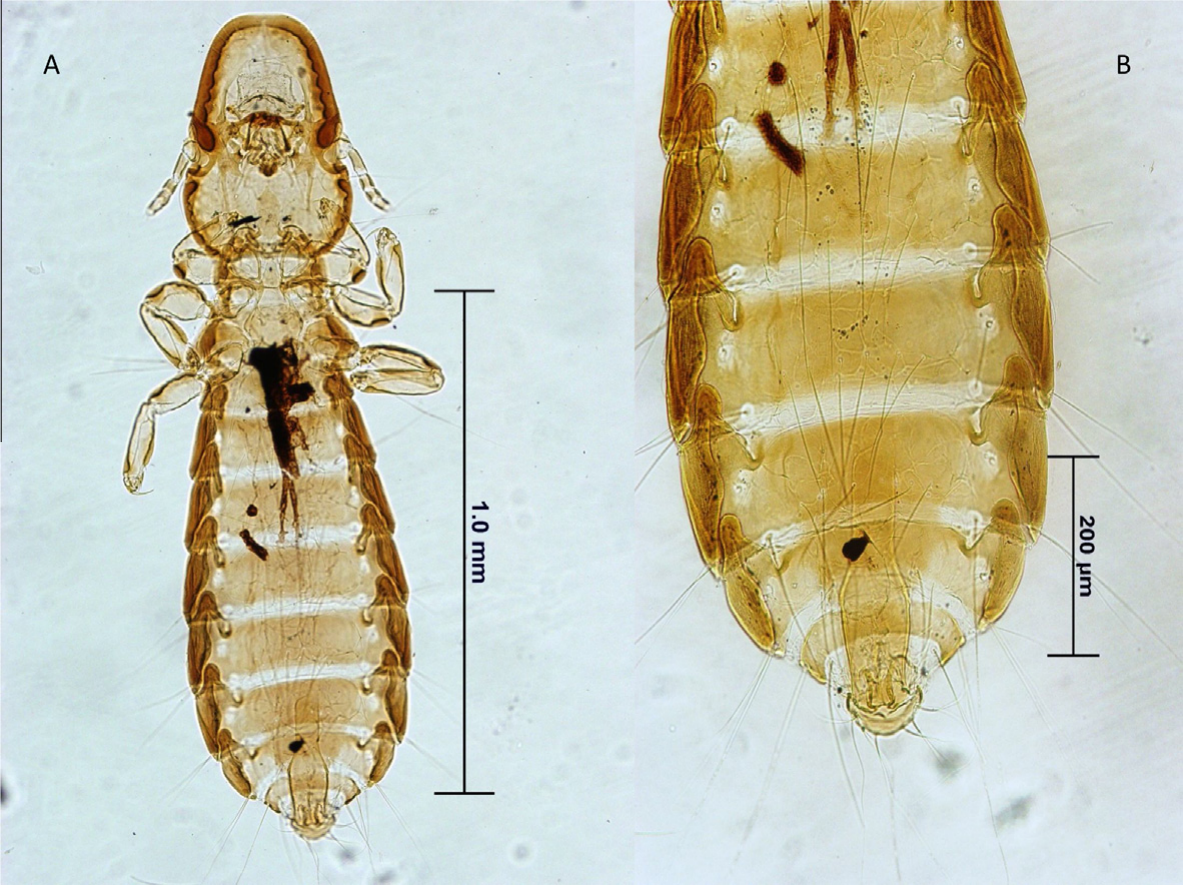
*Degeerialla nisus* from the common buzzard (*Bute buteo*). (A), Male, (B) male genitalia.

The detection of *D. nisus* (Giebel, 1866) (Fig. 1) on *Buteo buteo* (Common buzzard) demonstrated here represents a new host record for the lice fauna of the world. The following nine new lice records for Turkey were obtained: *Coloceras hilli* isolated from *Streptopelia decaocto* (Eurasian collared-dove); *I. plataleae* and *A. plataleae* isolated from *Platalea leucorodia* (Eurasian spoonbill); *F. suturalis* and *D. nisus* isolated from *B. buteo*; *Coloceras chinense* isolated from *Streptopelia senegalensis* (Laughing dove); *S. cursitans* isolated from *Athene noctua* (Little owl); *S. struthionis* isolated from *Struthio camelus* (Ostrich); and *G. pavonis* isolated from *Pavo cristatus* (Indian peafowl).

A large number of chewing lice of various species were detected on birds in the northwestern region of Turkey. Of the 29 species of chewing lice identified, 5 were associated with only a single host species. However, cosmopolitan species were also found, including *L. maximum*, which has been reported to parasitize 50 species of Falconiformes, and *S. lari*, which has been reported to parasitize 36 species of Charadriiformes (Price et al., 2003).

Of the 25 wild bird species examined, 13 species had not been previously evaluated for ectoparasite infestation in Turkey. These species include the European turtle doves, the laughing doves, the Dalmatian pelicans, the ostriches, the grey herons, the booted eagles, the Eurasian sparrowhawks, the little owls, the barn owls, the hooded crows, the western jackdaws*,* the Eurasian spoonbills and the willow warblers. In our study, no lice were detected on grey herons*,* barn owls, Eurasian sparrowhawks, hooded crows, western jackdaws or willow warblers, whereas all of the other species examined were infested with lice.

In this study, the infestation rate of migratory birds was notably higher than that of non-migratory birds (72.2% and 43.7%, respectively). In generalised studies of wild birds in Turkey, which lacked segregation based on migration, infestation rates of 25.0% (Dik, 2010), 41.4% (İnci et al., 2010b) and 35.4% (Dik et al., 2011b) were found. Additionally, studies conducted in the countries neighbouring Turkey showed infestation rates of 15.2% in Iran (Dik and Halajian, 2013), 10.7% in Bulgaria (Ilieva, 2005) and similar results in Russia (Lyakhova and Kotti, 2011). In addition, the species composition of the lice community and their hosts reported in our study were similar to those reported in Russia (Lyakhova and Kotti, 2011) and Bulgaria (Ilieva, 2005) but were markedly different than those reported in Iran (Dik and Halajian, 2013). This discrepancy may be due to geographical variations and the insufficient number of birds examined in the present study. In addition, infestation levels can vary according to habitat, the infestation rate in flocks of birds, the infestation of the nest, the general health of the birds and other environmental conditions (Poulin, 1991). Moreover, the higher infestation rates obtained in this study as compared to those conducted in other provinces in Turkey may have been the result of the high humidity levels in Bursa (Chen and Mullens, 2008; Bush et al., 2009).

Transmission of *D. nisus* between the Eurasian sparrowhawk, its normal host, and the Common buzzard was impossible because their reception at our hospital was separated by an interval of almost 1 year; the Common buzzard specimens were received in February 2011, while the Eurasian sparrowhawk was received in January 2012. In addition, special care was taken not to mix up the sampling tools. We are therefore confident that the Common buzzard represents a new host record for *D. nisus*.

In conclusion, we found that wild birds were infested by numerous lice species and that migratory birds were more significantly affected than non-migratory birds. Nine lice species, namely, *Coloceras hilli*, *C. chinense*, *I. plataleae*, *A. plataleae*, *F. suturalis*, *D. nisus, S. cursitans*, *S. struthionis* and *G. pavonis*, are reported here for the first time in Turkey. Additionally, we documented a new host report (*D. nisus* on *B. buteo*). Our findings indicate that Turkey is an important location in avian Phthirapteran research, and numerous lice species can be studied if a variety of bird species are examined on the flyways and/or important bird areas of Turkey.

## Figures and Tables

**Table 1:** Distribution of louse species according to their avian hosts.

*n*	Hosts	Common name	Chewing lice species	Abundance					
				Ni	M	F	N	T	MI
	COLUMBIFORMES								
	Columbidae								
1	*Streptopelia turtur*	Turtle dove	*Columbicola bacillus*	1	2	2	0	4	4.0
1	*Streptopelia senegalensis*	Laughing dove	*Coloceras chinense*^a^	1	5	1	0	6	6.0
2	*Streptopelia decaocto*	Eurasian collared-dove	*Columbicola bacillus*	1	1	1	0	2	2.0
			*Coloceras hilli*^a^	1	0	2	1	3	3.0
			*Coloceras piageti*	1	1	0	0	1	1.0

	CICONIIFORMES								
	Ciconiidae								
11	*Ciconia ciconia*	Stork	*Ciconiphilus quadripustulatus*	4	189	195	57	441	110.2
			*Neophilopterus incompletus*	3	6	0	0	6	2.0
			*Colpocephalum zebra*	1	1	2	0	3	3.0
			*Ardeicola ciconiae*	1	3	6	2	11	11.0

	PELECANIFORMES								
	Pelecanidae								
1	*Pelecanus crispus*	Dalmatian pelican	*Piagetiella titan*	1	5	5	3	13	13.0
			*Pectinopygus* sp.^c^	1	2	0	0	2	2.0
2	*Pelecanus onocrotalus*	Great white pelican	*Colpocephalum eucarenum*	1	1	0	0	1	1.0
			*Pectinopygus forficulatus*	1	167	212	65	444	444.0
			*Pectinopygus* sp.^c^	1	2	7	2	11	11.0
									
	Ardeidae								
1	*Ardea cinerea*	Grey heron	–	0	–	–	–	–	–
									
	Threskiornithidae								
1	*Platalea leucorodia*	Eurasian spoonbill	*Ibidoecus plataleaea*^a^	1	78	85	33	196	196.0
			*Ardeicola plataleae*^a^	1	2	1	0	3	3.0

	STRUTHIONIFORMES								
	Struthionidae								
1	*Struthio camelus* (farmed)	Ostrich	*Struthiolipeurus struthionis*^a^	1	9	11	3	23	23.0

	ACCIPITRIFORMES								
	Accipitridae								
14	*Buteo buteo*	Common buzzard	*Degeeriella fulva*	5	5	3	1	9	1.8
			*Degeeriella nisus*^a^^,^^b^	1	1	1	1	3	3.0
			*Degeeriella* sp.^c^	2	5	5	0	10	5.0
			*Craspedorrhynchus platystomus*	6	9	5	4	18	3.0
			*Colpocephalum nanum*	1	1	0	0	1	1.0
			*Falcolipeurus suturalis*^a^	1	1	1	0	2	2.0
			*Laemobothrion maximum*	3	4	1	1	6	2.0
2	*Buteo rufinus*	Long-legged buzzard	*Colpocephalum nanum*	1	2	1	3	6	6.0
			*Craspedorrhynchus platystomus*	1	1	7	2	10	10.0
1	*Circaetus gallicus*	Short-toed snake eagle	*Degeeriella leucopleura*	1	3	0	0	3	3.0
1	*Aquila pennatus*	Booted eagle	*Laemobothrion maximum*	1	1	0	0	1	1.0
1	*Accipiter nisus*	Sparrowhawk	–	0	–	–	–	–	–

	FALCONIFORMES								
	Falconidae								
1	*Falco tinnunculus*	Common kestrel	*Degeeriella rufa*	1	3	0	0	3	3.0

	PASSERIFORMES								
	Passeridae								
1	*Passer domesticus*	House sparrow	*Philopterus fringillae*	1	1	0	0	1	1.0
	Corvidae								
2	*Pica pica*	Eurasian magpie	–	0	–	–	–	–	–
1	*Corvus cornix*	Hooded crow	–	0	–	–	–	–	–
1	*Corvus monedula soemmerringii*	Western jackdaw	–	0	–	–	–	–	–
									
	Phylloscopidae								
1	*Phylloscopus trochilus*	Willow warbler	–	0	–	–	–	–	–

	CHARADRIIFORMES								
	Laridae								
14	*Larus cachinnans*	Caspian gull	*Saemundssonia lari*	4	5	1	2	8	2.0

	STRIGIFORMES								
	Strigidae								
1	*Athene noctua*	Little owl	*Strigiphilus cursitans*^a^	1	1	3	1	5	5.0
									
	Tytonidae								
4	*Tyto alba*	Barn owl	–	0	–	–	–	–	–

	GALLIFORMES								
	Phasianidae								
1	*Pavo cristatus* (farmed)	Indian peafowl	*Goniodes pavonis*^a^	1	3	3	0	6	6.0
1	*Alectoris chukar*	Chukar partridge	*Cuclotogaster heterographus*	1	1	5	0	6	6.0
			*Goniodes dispar*	1	2	7	1	10	10.0

68	Total			57	523	573	182	1278	24.7⁎

*n*: number of birds examined, Ni: number of birds infested, M: male, F: female, N: nymph, T: total, MI: mean intensity.

⁎ Considered for number of infested birds only.

a New geographic record for Turkey.

b New host record.

c Unspecified due to unsuccessful preparation.
